# Performance of artificial intelligence in predicting the prognossis of severe COVID-19: a systematic review and meta-analysis

**DOI:** 10.3389/fpubh.2024.1371852

**Published:** 2024-07-31

**Authors:** Chu Qin, Huan Ma, Mahong Hu, Xiujuan Xu, Conghua Ji

**Affiliations:** ^1^School of Public Health, Zhejiang Chinese Medical University, Hangzhou, China; ^2^Department of Critical Care Medicine, Tongde Hospital of Zhejiang Province, Hangzhou, China

**Keywords:** artificial intelligence, COVID-19, mortality, systematic reviews, meta-analyses

## Abstract

**Background:**

COVID-19-induced pneumonia has become a persistent health concern, with severe cases posing a significant threat to patient lives. However, the potential of artificial intelligence (AI) in assisting physicians in predicting the prognosis of severe COVID-19 patients remains unclear.

**Methods:**

To obtain relevant studies, two researchers conducted a comprehensive search of the PubMed, Web of Science, and Embase databases, including all studies published up to October 31, 2023, that utilized AI to predict mortality rates in severe COVID-19 patients. The PROBAST 2019 tool was employed to assess the potential bias in the included studies, and Stata 16 was used for meta-analysis, publication bias assessment, and sensitivity analysis.

**Results:**

A total of 19 studies, comprising 26 models, were included in the analysis. Among them, the models that incorporated both clinical and radiological data demonstrated the highest performance. These models achieved an overall sensitivity of 0.81 (0.64–0.91), specificity of 0.77 (0.71–0.82), and an overall area under the curve (AUC) of 0.88 (0.85–0.90). Subgroup analysis revealed notable findings. Studies conducted in developed countries exhibited significantly higher predictive specificity for both radiological and combined models (*p* < 0.05). Additionally, investigations involving non-intensive care unit patients demonstrated significantly greater predictive specificity (*p* < 0.001).

**Conclusion:**

The current evidence suggests that artificial intelligence prediction models show promising performance in predicting the prognosis of severe COVID-19 patients. However, due to variations in the suitability of different models for specific populations, it is not yet certain whether they can be fully applied in clinical practice. There is still room for improvement in their predictive capabilities, and future research and development efforts are needed.

**Systematic review registration:**

https://www.crd.york.ac.uk/prospero/ with the Unique Identifier CRD42023431537.

## 1 Introduction

The novel coronavirus disease 2019 (COVID-19) pandemic, caused by severe acute respiratory syndrome coronavirus 2 (SARS-CoV-2), has had a profound effect worldwide (1). As of December 2023, the World Health Organization has reported 773,119,173 COVID-19 cases (2). Currently, the medical community has conducted extensive research on the infection and pathogenesis mechanisms of COVID-19 (3). A series of measures have been implemented to control its spread and infection (4), such as the development of vaccines and the implementation of policies (5, 6).

While most individuals infected with SARS-CoV-2 experience either no or mild respiratory symptoms, a small percentage develop severe COVID-19 pneumonia or acute respiratory distress syndrome (ARDS). These cases can be life-threatening and necessitate intensive care or tracheal intubation (7–9). Studies have indicated that patients who undergo invasive mechanical ventilation are vulnerable to secondary infections, which can further increase the mortality rate (10). Additionally, a study has indicated that the infection fatality rate (IFR), the anticipated ratio between deaths and infections, among COVID-19 patients, exponentially increases with age (11). Furthermore, as the post-pandemic era unfolds, numerous previously infected individuals are expected to experience complications and sequelae, some of which may be severe and fatal (12). Consequently, accurate prognosis prediction is crucial for effectively managing these cases.

In recent years, there have been significant advancements in technology, leading to the continuous evolution of computer-aided techniques. These techniques have gradually developed into a diverse set of diagnostic and prognostic systems, with a particular focus on the field of medical imaging. These systems are designed to perform various tasks, including classification, regression, segmentation, and tracking (13). Artificial intelligence (AI) in medicine is evolving, reshaping medicine, and improving the experience of clinicians and patients (14–16). Research has demonstrated that artificial intelligence and machine learning can outperform clinical doctors in disease prediction on certain occasions (17).

In the context of COVID-19, a considerable number of studies have been conducted, employing artificial intelligence models for the purposes of diagnosis, treatment, and prediction (18–20). In intensive care respiratory medicine, artificial intelligence has made initial achievements in the prognostic prediction of diseases and has gradually become an auxiliary diagnostic tool for clinicians (21, 22). Recently, a study successfully developed a random forest model to predict hypotensive events in the Intensive Care Unit (ICU). The model exhibited an impressive sensitivity of 92.7%, enabling the prediction of these events up to 15 min in advance (23).

Multiple studies have documented the utilization and advancement of AI in prognostic prediction for critically-ill COVID-19 patients. However, there is a dearth of comprehensive evaluations regarding its effectiveness. Consequently, the true potential of current AI technology in clinical practice remains uncertain. To address this gap, our study aims to investigate how well AI methods provide reproducible prognostic predictions.

## 2 Methods

### 2.1 Protocol and registration

This study was performed in accordance with the Preferred Reporting Items for Systematic Reviews and Meta-Analyses (PRISMA) statement and involved a secondary analysis based on published researches (24). This study was registered in the International Prospective Register of Systematic Reviews (PROSPERO) database (Registration Number CRD42023431537). Ethics approval was not obtained for this study.

### 2.2 Database and search strategy

We searched PubMed,^1^ Web of Science,^2^ and Embase^3^ for all studies published before October 31, 2023, on the use of artificial intelligence techniques to predict the death of patients with severe COVID-19. Our search strategy included a combination of controlled vocabulary terms (NCBI’s MeSH terms) and free keywords. The keywords used encompassed terms such as “Critical Care,” “Artificial Intelligence,” and “COVID-19.” An example of the search strategy employed on PubMed can be found in Table 1.

**Table 1:** Literature search strategy on PubMed.

Search number	Query	Results
1	“Critical Care” [Mesh] OR “Critical care “[Title/Abstract] OR “Critical Illnesses” [Title/Abstract] OR “Illness, Critical” [Title/Abstract] OR “Illnesses, Critical” [Title/Abstract] OR “Critically Ill” [Title/Abstract] OR “Care, Critical” [Title/Abstract] OR “Intensive Care” [Title/Abstract] OR “Care, Intensive” [Title/Abstract] OR “Surgical Intensive Care” [Title/Abstract] OR “Care, Surgical Intensive” [Title/Abstract] OR “Intensive Care, Surgical” [Title/Abstract]	271,885
2	“Artificial Intelligence” [Mesh] OR “artificial intelligence” [Title/Abstract] OR “Intelligence, Artificial” [Title/Abstract] OR “Computational Intelligence” [Title/Abstract] OR “Intelligence, Computational” [Title/Abstract] OR “Machine Intelligence” [Title/Abstract] OR “Intelligence, Machine” [Title/Abstract] OR “Computer Reasoning” [Title/Abstract] OR “Reasoning, Computer” [Title/Abstract] OR “AI (Artificial Intelligence)” [Title/Abstract] OR “Computer Vision Systems” [Title/Abstract] OR “Computer Vision System” [Title/Abstract] OR “System, Computer Vision” [Title/Abstract] OR “Systems, Computer Vision” [Title/Abstract] OR “Vision System, Computer” [Title/Abstract] OR “Vision Systems, Computer” [Title/Abstract] OR “Knowledge Acquisition (Computer)” [Title/Abstract] OR “Acquisition, Knowledge (Computer)” [Title/Abstract] OR “Knowledge Representation (Computer)” [Title/Abstract] OR “Knowledge Representations (Computer)” [Title/Abstract] OR “Representation, Knowledge (Computer)” [Title/Abstract] OR “AI” [Title/Abstract]	222,730
3	“COVID 19” [Title/Abstract] OR “2019-nCoV Infection” [Title/Abstract] OR “2019 nCoV Infection” [Title/Abstract] OR “2019-nCoV Infections” [Title/Abstract] OR “Infection, 2019-nCoV” [Title/Abstract] OR “SARS-CoV-2 Infection” [Title/Abstract] OR “Infection, SARS-CoV-2” [Title/Abstract] OR “SARS CoV 2 Infection” [Title/Abstract] OR “SARS-CoV-2 Infections” [Title/Abstract] OR “2019 Novel Coronavirus Disease” [Title/Abstract] OR “2019 Novel Coronavirus Infection” [Title/Abstract] OR “COVID-19 Virus Infection” [Title/Abstract] OR “COVID 19 Virus Infection” [Title/Abstract] OR “COVID-19 Virus Infections” [Title/Abstract] OR “Infection, COVID-19 Virus” [Title/Abstract] OR “Virus Infection, COVID-19” [Title/Abstract] OR “COVID19” [Title/Abstract] OR “Coronavirus Disease 2019” [Title/Abstract] OR “Disease 2019, Coronavirus” [Title/Abstract] OR “Coronavirus Disease-19” [Title/Abstract] OR “Coronavirus Disease 19” [Title/Abstract] OR “Severe Acute Respiratory Syndrome Coronavirus 2 Infection” [Title/Abstract] OR “COVID-19 Virus Disease” [Title/Abstract] OR “COVID 19 Virus Disease” [Title/Abstract] OR “COVID-19 Virus Diseases” [Title/Abstract] OR “Disease, COVID-19 Virus” [Title/Abstract] OR “Virus Disease, COVID-19” [Title/Abstract] OR “SARS Coronavirus 2 Infection” [Title/Abstract] OR “2019-nCoV Disease” [Title/Abstract] OR “2019 nCoV Disease” [Title/Abstract] OR “2019-nCoV Diseases” [Title/Abstract] OR “Disease, 2019-nCoV” [Title/Abstract] OR “COVID-19 Pandemic” [Title/Abstract] OR “COVID 19 Pandemic” [Title/Abstract] OR “Pandemic, COVID-19” [Title/Abstract] OR “COVID-19 Pandemics” [Title/Abstract] OR “COVID-19” [Mesh]	340,152
4	#1 AND #2 AND #3	237

### 2.3 Inclusion criteria

(1) Research class: all articles in this study were published in English. (2) Study subjects: the participants were all patients aged >18 years diagnosed with COVID-19 and were either fully or partially treated in the ICU. (3) Patient grouping: deceased or surviving. (4) Outcome indicators: the actual number of deaths, actual number of survivors, at least two of AUC, sensitivity, specificity, accuracy, and F1-score were provided. (5) Study type: Cohort or case–control studies.

### 2.4 Exclusion criteria

(1) Studies with missing outcome indicators, unavailable data, or inconvertible data were excluded, as were (2) duplicated reports and (3) review reports. (4) The original articles were not included.

### 2.5 Screening of literature

After literature retrieval, repeated studies were excluded using the software. Two researchers read the titles and abstracts, screened them according to the inclusion and exclusion criteria, and obtained the full texts of the remaining literature. If the original text could not be obtained from the Internet, the author of the original text was contacted, and the full text was read and further screened.

### 2.6 Data extraction

Two researchers independently extracted the following literature data: author, publication year, study type, prediction model category, country, income level [high-income and non-high-income economies as defined by the World Bank (25)], total number of patients, actual number of dead patients, actual number of surviving patients, predicted number of dead patients, and predicted number of surviving patients (only the optimal model data of the external validation set were extracted; if not explicitly specified, the dataset provided in the article was assumed to be the validation set). After both researchers completed data extraction, the results were cross-checked, and discrepancies were discussed and finalized.

### 2.7 Risk of bias

Individual study bias was independently evaluated by two researchers using PROBAST 2019 (26), which includes two parts: risk of bias and applicability. The risk of bias section evaluates mainly participants, predictors, outcomes, and analyses. The applicability section evaluates the participants, predictors, and outcomes.

### 2.8 Evidence quality evaluation

In this study, we utilized the GRADE (Grading of Recommendations, Assessment, Development, and Evaluation) system to evaluate the credibility of the findings and assign a level of recommendation (27). It categorizes the evidence quality into four levels: high, moderate, low, and very low, considering factors such as quality of evidence, consistency of results, directness, precision, and potential bias.

### 2.9 Statistical methods

(1) Stata 16 software was used for statistical analysis. (2) The study was evaluated using sensitivity, specificity, 95% CI, and receiver operating characteristic (ROC) curves; comparisons were performed using forest plot descriptive statistics. (3) Literature heterogeneity was analyzed using *I*^2^ analysis, with *I*^2^ > 50% indicating heterogeneity of the results. (4) If no heterogeneity was found among the studies, the fixed effect model was used. If heterogeneity was evident among the studies, the random effects model was used for calculation; (5) heterogeneity survey: subgroup analysis was used to investigate heterogeneity; (6) sensitivity analysis was performed to detect the literature most affecting the effect size on the Diagnostic Odds Ratio (DOR) value; and (7) publication bias was detected using Egger’s test and presented using Deeks’ funnel plot.

## 3 Results

### 3.1 Literature screening

**Figure 1:**
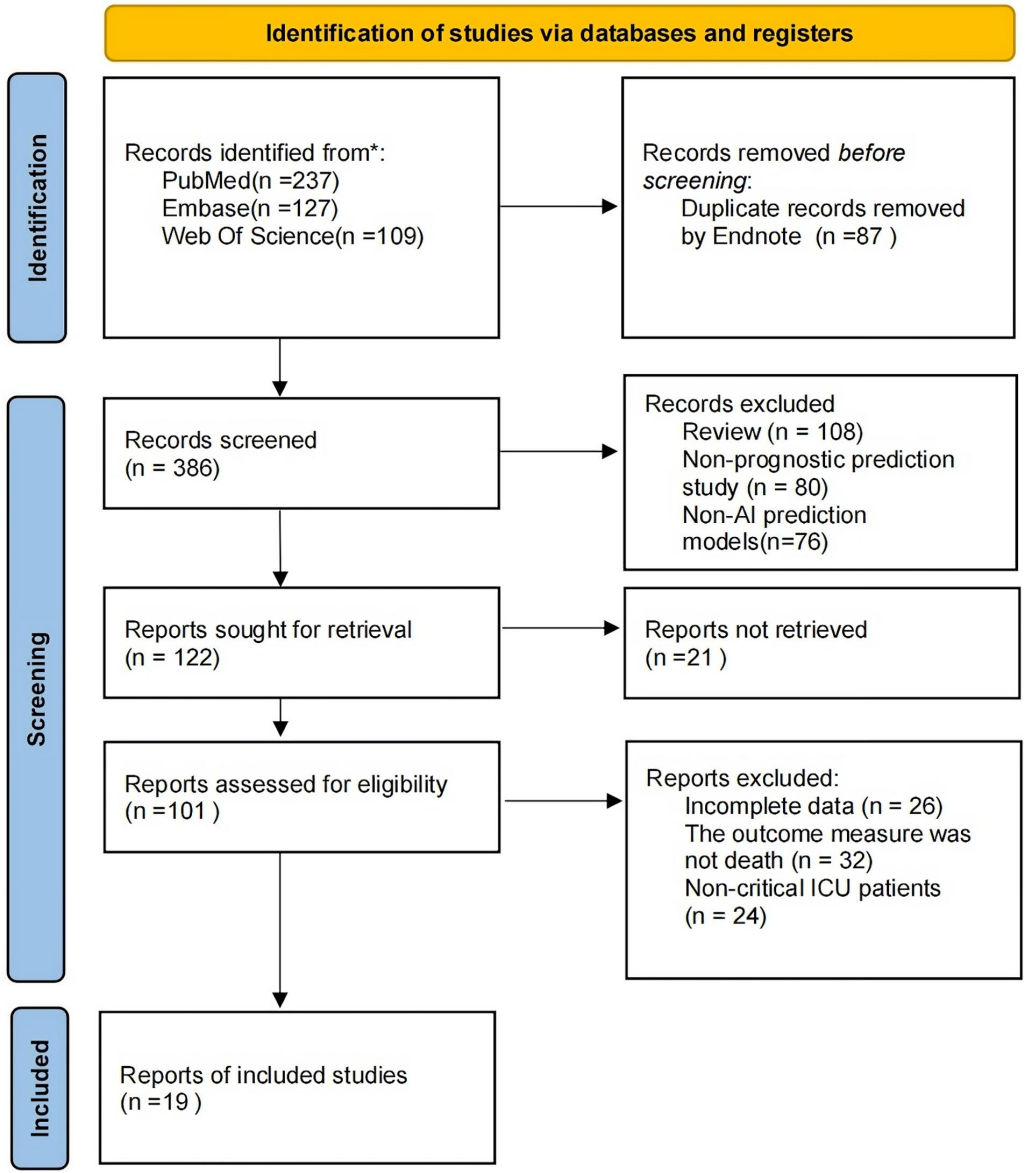
PRISMA flow diagram of study selection.

Figure 1 shows the PRISMA flow diagram of the study selection process. An exhaustive database search yielded 473 articles. Of these, 87 were eliminated through EndNote; the remaining 386 were screened for titles and abstracts. Following title and abstract screening, 285 publications were excluded, leaving 101 articles for full-text screening. The full texts of these 101 records were retrieved and reviewed for eligibility. For the reasons summarized in Figure 1, 82 articles were excluded. Ultimately, 19 studies were included in our Meta-analysis (22, 28–44).

### 3.2 Study characteristics

Table 2 lists the datasets extracted from selected articles. Briefly, they were published between 2021 and 2022 in Europe (*n* = 9), Asia (*n* = 4), North America (*n* = 3), South America (*n* = 1), and Africa (*n* = 1). Fifteen of the 18 studies were retrospective, in which past samples/images were evaluated; the remaining three articles were prospective. Clinical data were the most commonly used predictors (*n* = 12); a combination of clinical and imaging data were used to predict severe COVID-19 mortality (*n* = 8). The included studies had two main categories of AI models: machine learning (*n* = 9) and deep learning (*n* = 9).

**Table 2:** Characteristics and extracted data from included studies.

Author	Year	Country	Income levels	Region	Study design	Parameters of model	Type	Predictive factors	Survivors	Deaths	Total
Walston, S. L.	2022	Japan	High-income	Asia	Retrospective	ID/CD/ID & CD	DL	Chest radiographs (ID)	230	43	273
								Gender, age, smoking history, BMI, medical history, laboratory data (CD)			
Vagliano, I.	2022	The Netherlands	High-income	Europe	Prospective	CD	ML	Demographic data, physiological data, diagnoses (reason for admission and comorbidities)	1894	796	2,690
Puhr-Westerheide, D.	2022	Germany	High-income	Europe	Retrospective	CD	DL	SOFA Score on Admission, Age	53	36	89
Pezoulas, V. C.	2022	Greece	High-income	Europe	Retrospective	CD	ML	607 clinical features	595	70	665
Munera, N.	2022	Colombia	Non-high-income	South America	Prospective	ID/CD/ID & CD	ML	Chest radiographs (ID)	47	22	69
								Sociodemographic variables, comorbid conditions, symptoms, vital signs on hospital admission and treatments received during the hospitalization (CD)			
Klén, R.	2022	Finland	High-income	Europe	Retrospective	CD	ML	Age, Sex, Hemoglobin, Platelet Count, Eosinophils, Lymphocytes, Neutrophils, Monocytes, C-Reactive Protein, Creatinine, Lactate Dehydrogenase, Aspartate aminotransferase, Alanine aminotransferase, Total bilirrubin, Serum Sodium, Serum Potassium, Glucose, Prothrombin time, Fibrinogen, Dimer	4,352	756	5,108
Elghamrawy, S. M.	2022	Egypt	Non-high-income	Africa	Retrospective	ID & CD	DL	CT (ID)	9,352	897	10,249
								Clinical features (e.g., sex), laboratory data (e.g., D-Dimer) (CD)			
Di Napoli, A.	2022	Italy	High-income	Europe	Retrospective	ID/ID & CD	DL	CT (ID)	223	45	269 (ID&CD)
								Demographics, comorbidities, symptoms (CD)	243	47	290 (ID)
Chrzan, R.	2022	Poland	High-income	Europe	Retrospective	ID	DL	High-resolution computed tomography (HRCT)	699	105	804
Cheng, J.	2022	China	Non-high-income	Asia	Retrospective	ID/CD/ID & CD	DL	Chest radiographs (ID)	59	49	108
								Demographic, clinical, and laboratory variables			
Chamberlin, J. H.	2022	United States	High-income	North America	Retrospective	ID & CD	DL	CT (ID)	228	14	242
								Age, BMI, Symptom days, PCR-Imaging A, Sex, Female, Male, Ethnicity, Black, Hispanic, Other, White, Prior Structural Lung disease, History of Cancer, Smoking History, Hypertension, Diabetes, CHF, CKD, Autoimmune disease, HIV (CD)			
Calvillo-Batllés, P.	2022	Spain	High-income	Europe	Prospective	ID & CD	ML	Chest radiographs (ID)	74	14	88
								Demographics, clinical and laboratory variables (CD)			
Wanyan, Tingyi	2021	United States	High-income	North America	Retrospective	CD	DL	Demographics, lab test results, vital signs, comorbid diseases	283	98	381
Pezoulas, V. C.	2021	Greece	High-income	Europe	Retrospective	CD	ML	Demographic information, comorbidities, laboratory tests (e.g., C-reactive protein), therapies (corticosteroids and antiviral agents), cytokines and interleukins measurements at four time intervals	178	36	214
Khan, I. U.	2021	Saudi Arabia	High-income	Asia	Retrospective	CD	DL	Demographic, Hospital Attribute, Symptoms, Chronic Disease	97,941	5,947	103,888
Jamshidi, E.	2021	Iran	Non-high-income	Asia	Retrospective	CD	ML	Gender, Age, Blood Urea Nitrogen, Creatinine, INR, Albumin, WBC, Neutrophil count, Lymphocyte count, RDW, MCH, Neurological disorders, Cardiovascular disorders, Respiratory disorders	105	158	263
Hou, W.	2021	United States	High-income	North America	Retrospective	CD	ML	Age, Heart Failure, LDH, CRP, Hypertension, Immunosuppression, CRP, LDH, Spo, Heart Failure, Smoking, SBP	553	82	635
Chassagnon, G.	2021	France	High-income	Europe	Retrospective	ID & CD	ML	Imaging from the disease regions (5 features), lung regions (5 features) and heart features (5 features), biological and clinical data (6 features: age, sex, high blood pressure (HBP), diabetes, lymphocyte count and CRP level) and image indexes (2 features: disease extent and fat ratio), 23 biomarker consisted	149	8	157

DL, Deep learning; ML, Machine learning; ID, Image data; CD, Clinical data.

### 3.3 Risk of bias

Bias assessment included the risk of bias and applicability sections. The results of the qualitative assessment of the included studies are shown in Table 3.

**Table 3:** Assessment of bias by PROBAST.

		Risk of bias				Applicability			Overall assessment	
Author	Year	Participants	Predictors	Outcome	Analysis	Participants	Predictors	Outcome	Risk of bias	Applicability
Walston, S. L.	2022	−	?	−	?	?	−	−	?	?
Vagliano, I.	2022	−	?	−	−	−	−	−	?	−
Puhr-Westerheide, D.	2022	−	?	?	−	−	−	−	?	−
Pezoulas, V. C.	2022	−	−	−	−	−	−	−	−	−
Munera, N.	2022	−	−	−	−	−	−	−	−	−
Klén, R.	2022	−	?	−	−	−	−	−	?	−
Elghamrawy, S. M.	2022	−	?	+	−	−	−	−	+	−
Di Napoli, A.	2022	−	?	−	+	−	−	−	+	−
Chrzan, R.	2022	−	?	−	+	−	−	−	+	−
Cheng, J.	2022	−	?	−	+	−	−	−	+	−
Chamberlin, J. H.	2022	−	−	−	−	−	−	−	−	−
Calvillo-Batllés, P.	2022	−	−	−	−	−	−	−	−	−
Wanyan, Tingyi	2021	−	?	−	+	−	−	−	+	−
Pezoulas, V. C.	2021	−	−	−	−	−	−	−	−	−
Khan, I. U.	2021	−	−	−	−	−	−	−	−	−
Jamshidi, E.	2021	−	?	−	?	−	−	−	?	−
Hou, W.	2021	−	?	−	?	−	−	−	?	−
Chassagnon, G.	2021	−	?	−	−	−	−	−	?	−

For bias risk assessment, six articles (22, 31, 37, 38, 40, 41) were determined to have a low risk, demonstrating robust methodologies and transparent reporting. Conversely, five articles (33–36, 39) were assigned a high risk of bias due to several factors. These included a lack of reporting on the appropriate handling of missing data and the determination of outcomes without prior knowledge of the predictive factor information. These issues pose a potential threat to the validity of our results. The risk levels for seven articles (28–30, 32, 42–44) remained ambiguous owing to insufficient information. The articles did not explicitly state whether the assessment of predictive factors was conducted without knowledge of the outcome data and whether issues such as model overfitting and optimistic model performance could be explained. This underscores the need for more comprehensive reporting.

With respect to the applicability assessment, the majority of articles (17 out of 18) were deemed to be of low risk, suggesting that their findings are likely relevant and applicable to the research context. However, one article (28) received an unclear rating due to insufficient evidence to confirm that the included participants and study setting aligned with the research question.

### 3.4 Sensitivity, specificity, and ROC curve

**Figure 2:**
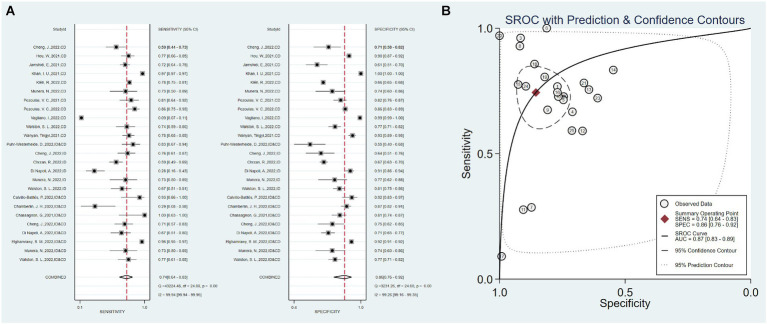
Overall sensitivity, specificity, and ROC curve. **(A)** Overall sensitivity and specificity, **(B)** overall ROC curve.

The 18 studies, including 25 AI prediction models, had an overall sensitivity of 0.74 (0.64–0.83, *I*^2^ = 99.94%) and a specificity of 0.86 (0.76–0.92, *I*^2^ = 99.26%) using clinical data, imaging data and the combination of clinical and imaging data. The overall AUC was 0.87 (0.83–0.89) (Figure 2).

#### 3.4.1 Clinical data

**Figure 3:**
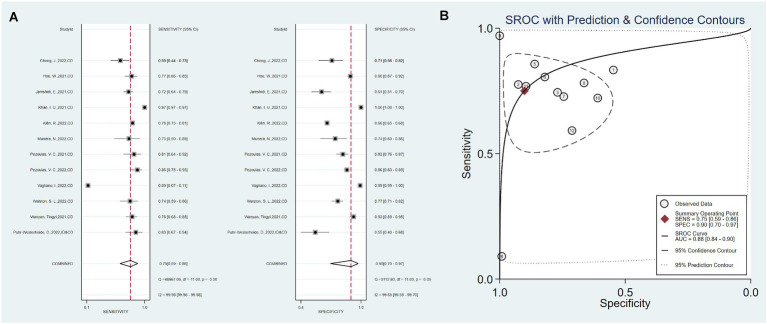
Sensitivity, specificity, and ROC curve based on clinical data. **(A)** Sensitivity and specificity based on clinical data model, **(B)** ROC curve based on clinical data.

Twelve studies used only clinical data as predictors, with a total sensitivity of 0.75 (0.59–0.86) and *I*^2^ = 99.98%. The total specificity was 0.90 (0.70–0.97), *I*^2^ = 99.65% (Figure 3). The overall AUC was 0.88 (0.84–0.90).

#### 3.4.2 Imaging data

**Figure 4:**
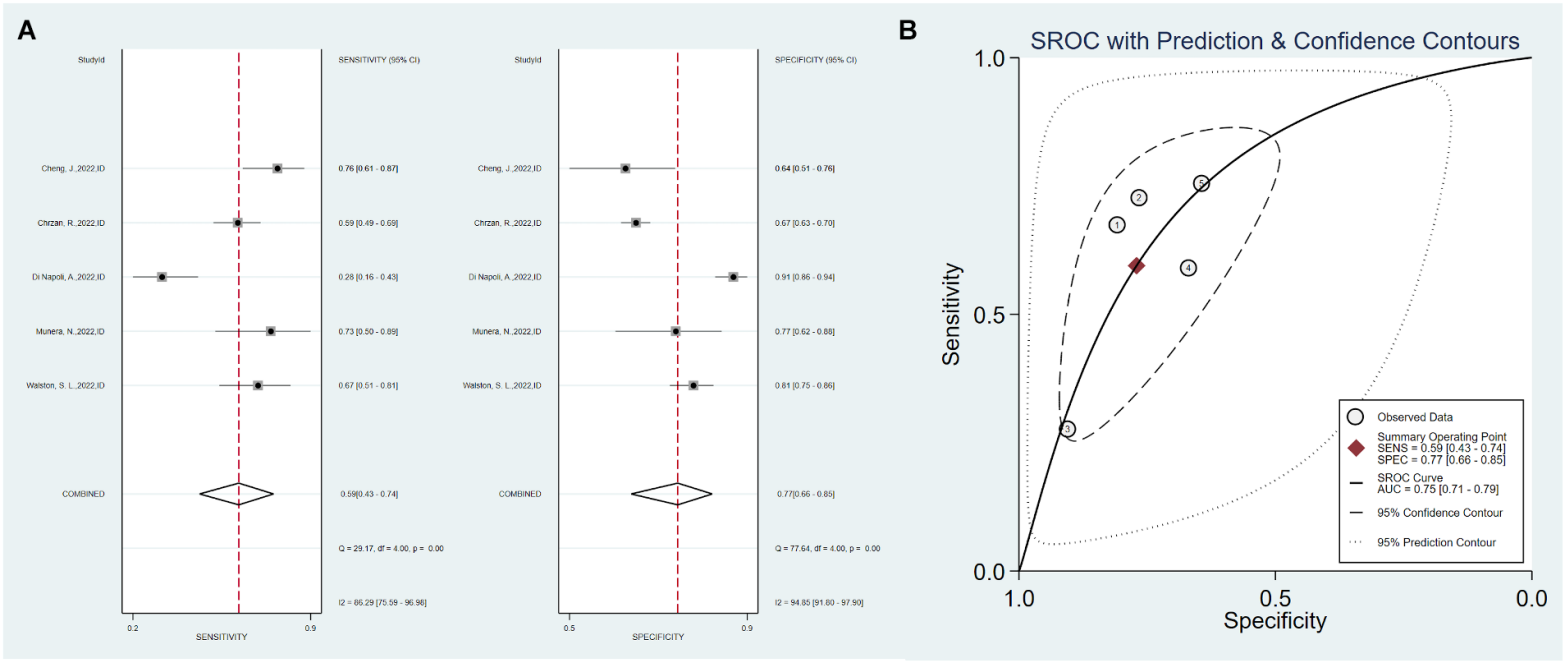
Sensitivity, specificity, and ROC curve based on imaging data. **(A)** Sensitivity and specificity based on imaging data model, **(B)** ROC curve based on imaging data.

Five studies used only imaging data as predictors, with a total sensitivity of 0.59 (0.43–0.47) and *I*^2^ = 86.29%. The total specificity was 0.77 (0.66–0.85), *I*^2^ = 94.85%. The overall AUC was 0.75 (0.71–0.79) (Figure 4).

#### 3.4.3 Combining clinical and imaging data

**Figure 5:**
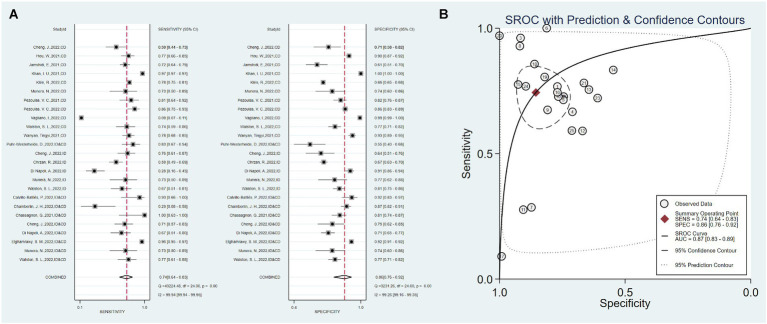
Sensitivity, specificity, and ROC curve based on combined clinical and imaging data. **(A)** Sensitivity and specificity based on combined data, **(B)** ROC curve based on combined data.

Eight studies combined clinical data with imaging data, with a total sensitivity of 0.81 (0.64–0.91) and *I*^2^ = 95.18%. The total specificity was 0.77 (0.71–0.82), *I*^2^ = 98.99%. The overall AUC was 0.88 (0.85–0.90) (Figure 5).

### 3.5 Fagan plot

**Figure 6:**
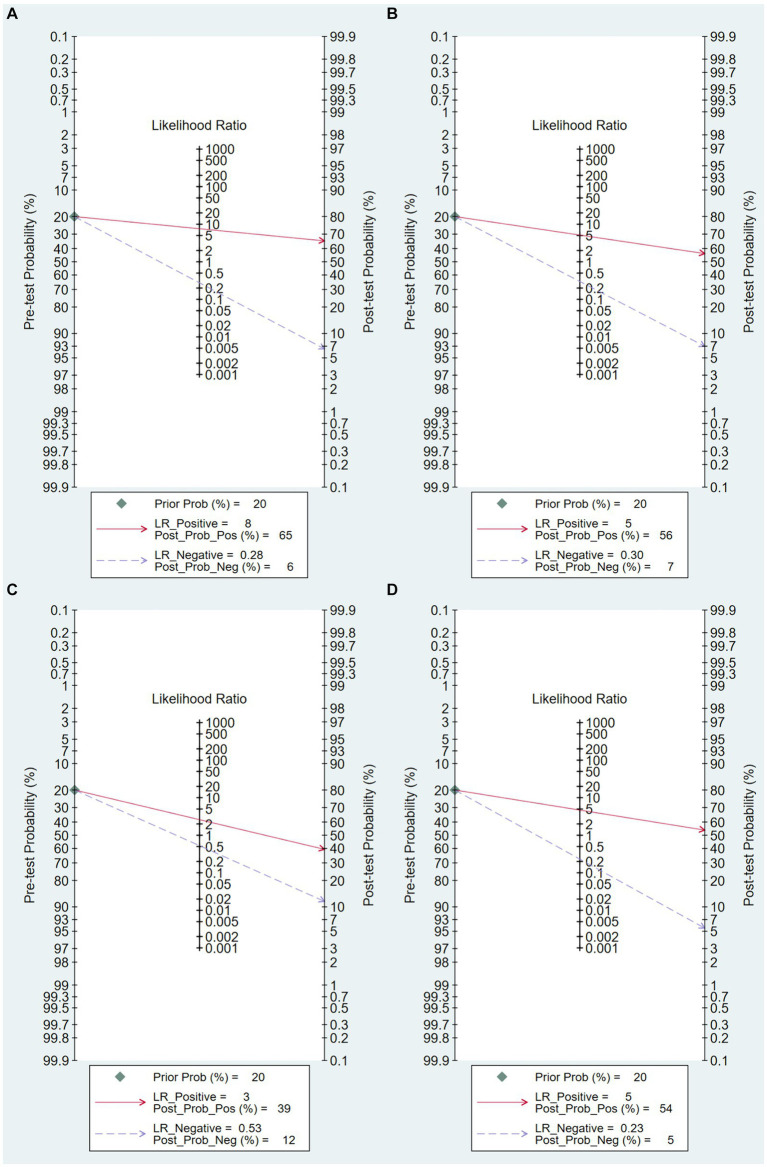
Fagan Plot. **(A)** Overall Fagan plot, **(B)** Fagan plot based on clinical data model, **(C)** Fagan plot based on imaging data model, **(D)** Fagan plot based on combined clinical and imaging data model.

The Fagan’s nomogram was used to evaluate the diagnostic performance of the AI model in predicting mortality in severe COVID-19 cases. In Figure 6A, The pre-test probability, or the anticipated probability of mortality prior to the test results, was set at 20%. The likelihood ratios for positive and negative results for all the included articles were 5 and 0.30, respectively. These values yielded post-test probabilities of 56% for positive results and 7% for negative results.

#### 3.5.1 Clinical data

Figure 6B illustrates that, given an initial expected mortality probability of 20% prior to testing, a prediction model solely utilizing clinical data as predictors yielded likelihood ratios of 8.0 for positive results and 0.28 for negative results. These values yielded post-test probabilities of 65% for positive results and 6% for negative results.

#### 3.5.2 Imaging data

As shown in Figure 6C, under the presumption of a 20% mortality probability prior to testing, a prediction model solely utilizing imaging data as predictors yielded likelihood ratios of 3 for positive results and 0.58 for negative results. These values yielded post-test probabilities of 39% for positive results and 12% for negative results.

#### 3.5.3 Combining clinical and imaging data

As shown in Figure 6D, with an assumed pre-test mortality probability of 20%, a prediction model that used a combination of clinical and radiographic data as predictors yielded likelihood ratios of 3 for positive results and 0.58 for negative results. These values yielded post-test probabilities of 39% for positive results and 12% for negative results.

### 3.6 Subgroup analyses

Meta-regression and subgroup analyses we conducted to explore the effects of different model types, economic income levels, study design methods, and settings in which all patients came from the ICU on the prediction power.

**Figure 7:**
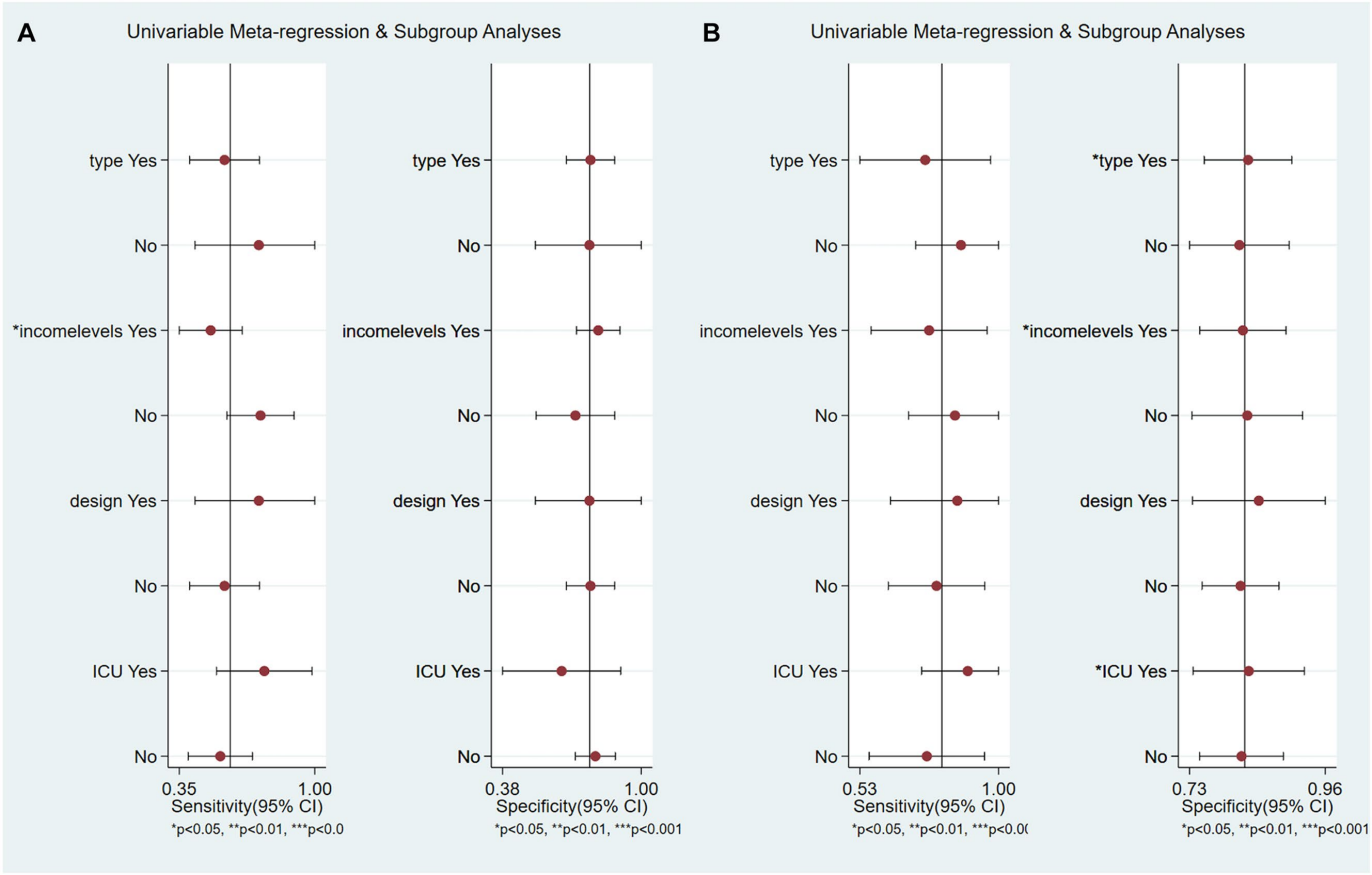
Meta-regression and subgroup analysis. **(A)** Based on imaging data prediction models, **(B)** Based on combination of imaging and clinical data prediction models.

The results show that, within the context of the imaging data prediction model, high-income economies exhibited heightened prediction sensitivity (*p* < 0.05). Conversely, as depicted in Figure 7A, the model type, study design, and ICU status did not show significant disparities. Within the combined clinical and radiological prediction model, we observed several significant differences. Notably, when all patients were exclusively from the Intensive Care Unit, as well as in the case of artificial intelligence prediction models utilizing deep learning and models originating from high-income economies, a higher predictive specificity was demonstrated (*p* < 0.05) as illustrated in Figure 7B.

### 3.7 Sensitivity analysis

A sensitivity analysis was conducted to explore the robustness and consistency of the results. As shown in Table 4, the overall value of the combined effect of DOR did not change significantly after removing them individually from the model; the stability was good. However, in the model based on imaging data, after excluding the studies by Walston et al. (28) and Chrzan et al. (35) the overall *I*^2^ changed from 55.6 to 29.30 and 2.70%, respectively, indicating that these two studies may be a source of heterogeneity (Tables 5, 6). In addition, among the eight studies based on the combination of clinical and imaging models, the overall heterogeneity was reduced from 96.2 to 58.6% after the exclusion of the study by Elghamrawy et al. (33) indicating that it may be the main source of heterogeneity (Table 7).

**Table 4:** Sensitivity analysis of included 25 studies (total).

Study omitted	Estimate (DOR)	[95% confidence interval]		*I* ^2^
Walston, S. L., 2022, ID & CD	14.401	7.691	26.966	95.70%
Munera, N., 2022, ID & CD	14.561	7.823	27.100	95.70%
Elghamrawy, S. M., 2022, ID & CD	10.741	7.212	15.996	87.20%
Di Napoli, A., 2022, ID & CD	14.929	7.980	27.931	95.70%
Cheng, J., 2022, ID & CD	14.641	7.838	27.349	95.70%
Chassagnon, G., 2021, ID & CD	13.664	7.410	25.198	95.70%
Chamberlin, J. H., 2022, ID & CD	15.147	8.167	28.095	95.70%
Calvillo-Batllés, P., 2022, ID & CD	13.239	7.176	24.423	95.70%
Walston, S. L., 2022, ID	14.558	7.765	27.291	95.70%
Munera, N., 2022, ID	14.492	7.786	26.973	95.70%
Di Napoli, A., 2022, ID	15.086	8.096	28.112	95.60%
Chrzan, R., 2022, ID	15.291	8.202	28.506	95.30%
Cheng, J., 2022, ID	14.812	7.935	27.646	95.70%
Puhr-Westerheide, D., 2022, CD	14.727	7.908	27.429	95.70%
Wanyan, Tingyi, 2021, CD	13.513	7.254	7.254	95.60%
Walston, S. L., 2022, CD	14.485	7.732	27.134	95.70%
Vagliano, I., 2022, CD	14.425	7.633	27.259	95.70%
Pezoulas, V. C., 2022, CD	13.619	7.303	25.398	95.60%
Pezoulas, V. C., 2021, CD	14.048	7.528	26.214	95.70%
Munera, N., 2022, CD	14.561	7.823	27.100	95.70%
Klén, R., 2022, CD	15.170	7.514	30.626	95.30%
Khan, I. U., 2021, CD	11.091	6.192	19.865	95.20%
Jamshidi, E., 2021, CD	15.092	8.047	28.304	95.60%
Hou, W., 2021, CD	13.811	7.361	25.911	95.60%
Cheng, J., 2022, CD	15.092	8.103	28.107	95.60%
Combined	14.182	7.746	25.965	95.50%

**Table 5:** Sensitivity analysis of included 12 studies (based on clinical data).

Study omitted	Estimate (DOR)	[95% confidence interval]		*I* ^2^
Puhr-Westerheide, D., 2022	18.056	9.326	34.958	92.50%
Wanyan, Tingyi, 2021	14.668	7.828	27.484	91.00%
Walston, S. L., 2022	17.578	8.941	34.559	92.50%
Vagliano, I., 2022	17.538	8.733	35.220	92.50%
Pezoulas, V. C., 2022	15.073	7.935	28.630	91.60%
Pezoulas, V. C., 2021	16.356	8.444	31.685	92.40%
Munera, N., 2022	17.600	9.111	34.000	92.50%
Klén, R., 2022	19.495	8.948	42.478	91.30%
Khan, I. U., 2021	11.621	7.113	18.986	87.10%
Jamshidi, E., 2021	19.299	9.798	38.013	91.90%
Hou, W., 2021	15.578	8.087	30.009	91.60%
Cheng, J., 2022	19.077	9.865	36.891	92.20%
Combined	16.431	8.820	30.612	91.80%

**Table 6:** Sensitivity analysis of included five studies (based on imaging data).

Study omitted	Estimate (DOR)	[95% Confidence Interval]		*I* ^2^
Walston, S. L., 2022	3.971	2.587	6.096	29.30%
Munera, N., 2022	4.546	2.696	7.667	59.40%
Di Napoli, A., 2022	5.446	2.902	10.220	66.10%
Chrzan, R., 2022	6.081	3.988	9.274	2.70%
Cheng, J., 2022	4.886	2.674	8.928	64.80%
Combined	4.922	3.015	8.036	55.60%

**Table 7:** Sensitivity analysis of included eight studies (based on the combined data).

Study omitted	Estimate (DOR)	[95% confidence interval]		*I* ^2^
Walston, S. L., 2022	20.199	3.096	131.805	96.70%
Munera, N., 2022	21.095	3.522	126.358	96.90%
Elghamrawy, S. M., 2022	8.666	4.504	16.677	58.60%
Di Napoli, A., 2022	22.733	3.971	130.150	96.00%
Cheng, J., 2022	21.398	3.489	131.249	96.70%
Chassagnon, G., 2021	16.006	2.824	90.714	97.00%
Chamberlin, J. H., 2022	24.424	4.358	136.876	96.60%
Calvillo-Batllés, P., 2022	14.325	2.479	82.778	97.00%
Combined	18.504	3.615	94.713	96.50%

### 3.8 Publication bias

**Figure 8:**
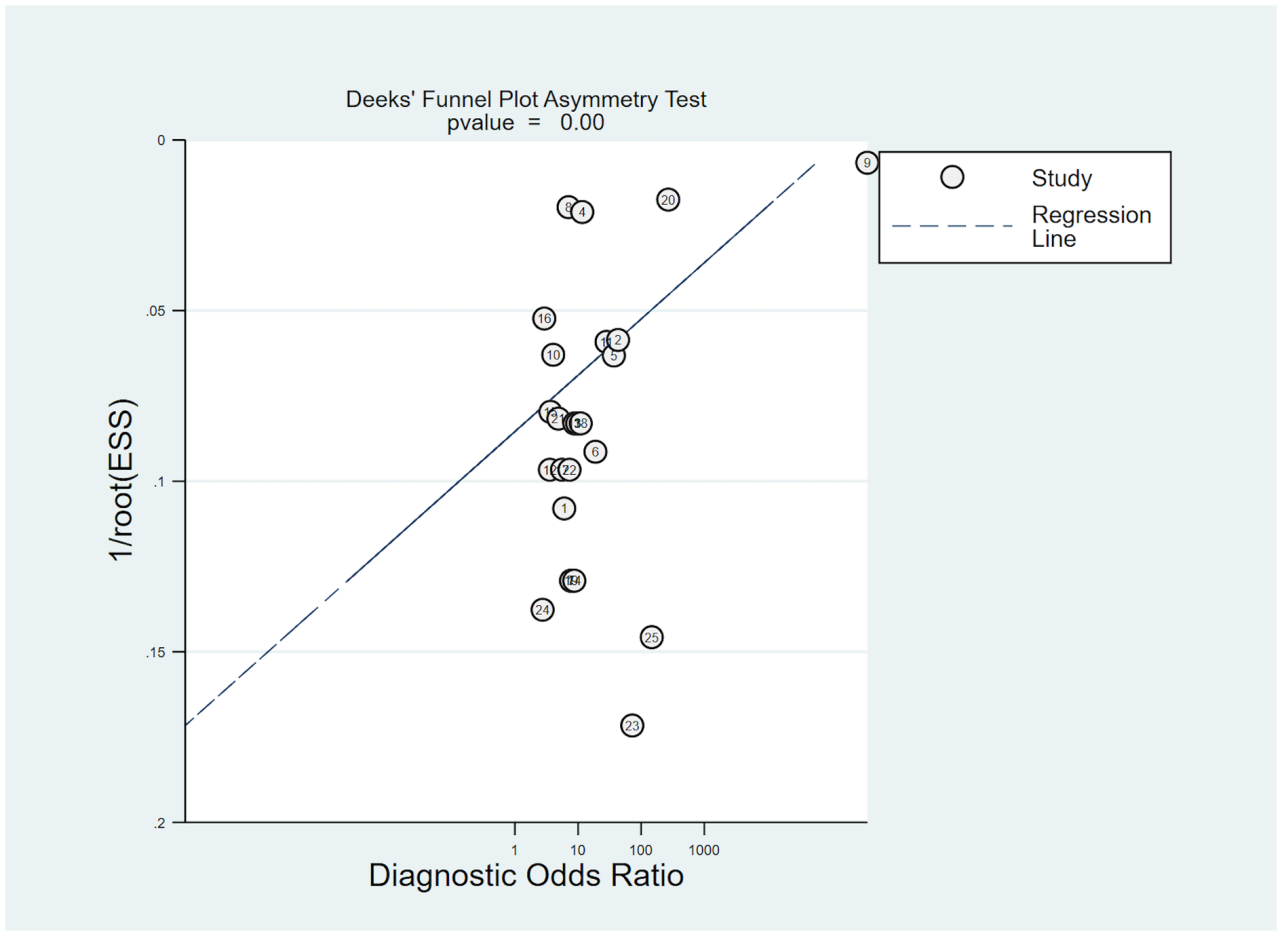
Deek’s funnel plot was used to evaluate publication bias.

The Deeks’ Funnel Plot Asymmetry Test, as depicted in Figure 8, was utilized to investigate the presence of publication bias. The studies, represented by individual data points, are not symmetrically distributed around the regression line (*p* < 0.001), suggesting the presence of publication bias or other small-study effects in the meta-analysis.

### 3.9 Evidence quality evaluation

The aforementioned four important outcomes in this meta-analysis were evaluated using the GRADE system. The evidence quality level for each outcome was found to be very low Table 8. Since all the included studies in this research had observational designs, the initial quality rating of the studies was assessed as “low.” The final overall level of evidence was rated as “very low,” which may decrease the credibility of any recommendations.

**Table 8:** The GRADE evidence quality for each outcome.

Model type	Design	Number of models	Sensitivity	Specificity	ROC-AUC	Risk of bias	Inconsistency	Indirectness	Imprecision	Publication bias	Evidence quality
Overall study models	Cohort studies	25	0.74	0.86	0.87	No degradation	Downgrade^1^	No degradation	No degradation	Downgrade^2^	Very low
Clinical data-based models	Cohort studies	12	0.78	0.9	0.88	No degradation	Downgrade^1^	No degradation	No degradation	No degradation	Very low
Imaging data-based models	Cohort studies	5	0.59	0.77	0.75	No degradation	Downgrade^1^	No degradation	No degradation	No degradation	Very low
Clinical and imaging data combined models	Cohort studies	8	0.81	0.83	0.88	No degradation	Downgrade^1^	No degradation	No degradation	No degradation	Very low

1 Significant heterogeneity observed (*I*^2^ > 70%).

2 Publication bias detected.

## 4 Discussion

Since the emergence of COVID-19 in 2019, its global spread of novel coronavirus infection has caused significant disruptions in mortality patterns worldwide (45, 46). The ongoing 2019 coronavirus disease pandemic has prompted substantial efforts to prevent and treat COVID-19, leading to remarkable achievements (47–50). The World Health Organization ceased considering COVID-19 a global health emergency in May 2023. Despite these advancements, SARS-CoV-2 continues to persist, evolve, and threaten human life (51). Long COVID-19 is our big challenge (52, 53). Severe COVID-19 presents an ongoing and substantial risk to individual health and well-being, with mortality rates as high as 49% among critically ill individuals (54). Research exploring AI as an assistive tool for COVID-19 in this environment has gained prominence over the past 3 years (55–57). This study investigated this important topic.

AI is increasingly used in medicine (14). AI can autonomously search and extract intricate task-specific features, offering the advantage of cost-effectiveness (56, 58). This systematic review and meta-analysis meticulously synthesized the available evidence regarding the performance of AI prediction models for severe COVID-19 mortality. In the included literature, the clinical data encompassed elements including demographic information (such as age and sex), comorbidities (hypertension, stroke, atrial fibrillation, etc.), laboratory data (albumin, hemoglobin, sodium, potassium, etc.), and imaging data, typically consisting of CT scans, X-rays, and their associated parameters. Integrating clinical and imaging data is pivotal in precision medicine and large-scale research programs, enabling a comprehensive understanding of disease patterns and facilitating targeted and effective interventions (59, 60). The meta-analysis encompassed 25 prediction models, showing commendable sensitivity and specificity, with an area under the curve (AUC) of 0.74 (0.64–0.83) and 0.86 (0.76–0.92), respectively. Furthermore, the Fagan plot demonstrated commendable positive post-test probability (56%) and negative post-test probability (7%). This indicates that when the AI model provided a positive prediction, the probability of death increased from the pre-test probability of 20 to 56%. Conversely, negative predictions reduced the mortality rate by 7%. This substantial difference underscores the potential utility of AI models in identifying high-risk patients who may require more intensive care or intervention as well as their effectiveness in identifying patients with a lower risk of death. Overall, the results suggest that the AI prediction models hold promise for predicting severe COVID-19 mortality rates.

However, 25 studies included in our analysis exhibited significant heterogeneity. To explore the sources of heterogeneity, we conducted sensitivity and subgroup analyses. The overall results showed that despite Khan et al. (41) having a significantly larger sample size than the others, the exclusion of this study did not result in a significant change in the diagnostic odds ratio value and its heterogeneity. This suggests that an imbalance in the dataset was not the primary source of heterogeneity. We also conducted a sensitivity analysis of different subgroups of predictive factors. We found that, in models based on radiological data, the exclusion of the study by Chrzan et al. (35) resulted in a substantial decrease in *I*^2^ to 2.7% and an increase in the DOR value. This could be attributed to the unique nature of the predictive data used in this study, which were derived from High-Resolution Computed Tomography (HRCT) and its specific parameters such as absolute inflammation volume and absolute consolidation volume (ACV), as opposed to conventional CT scans or chest X-rays. We conjecture that HRCT, as a relatively novel diagnostic instrument, may not be universally applicable to all patient populations. This potential limitation could contribute to the observed decrease in the DOR value.

Additionally, we conducted subgroup analyses based on different model types, income levels, types of study designs, and ICU states. Our results indicate that studies employing deep learning, originating from high-income economies, or involving patients exclusively in the ICU, often exhibit higher specificity. A plausible explanation for this could be the urgent need for ICU patients and those in high-income economies to avoid death, which might cause the models to lean more toward predicting a higher risk of mortality. This could potentially enhance the model’s ability to correctly identify true-negative cases, thereby increasing specificity. However, it is important to note that although this might improve the model’s performance in an ICU setting, it may not necessarily work in other contexts. Furthermore, our findings suggest that studies employing deep learning models demonstrate higher specificity. This can be attributed to the inherent capabilities of deep learning models, which are adept at capturing complex nonlinear relationships in high-dimensional data. This allows them to discern subtle patterns that may be overlooked by traditional statistical models, thereby enhancing their ability to correctly identify true-negative cases, and consequently, increasing their specificity. However, it is important to note that although deep learning models can offer improved performance, their effectiveness is heavily dependent on the quality and diversity of the training data. Therefore, future studies should focus on ensuring the collection of comprehensive, high-quality datasets that capture a wide range of patient characteristics and clinical scenarios. Moreover, given the black-box nature of deep learning models, efforts should be made to improve their interpretability (61).

This study had limitations. First, the sample size of the included studies was relatively small, and only English literature was considered, potentially introducing language and publication biases. The exclusion of literature in other languages, including German, Japanese, and Korean, may have influenced the findings. Second, the funnel plot analysis revealed an asymmetric funnel distribution, suggesting the presence of publication bias. This bias could affect the overall interpretation of the results and the generalizability of the findings. Furthermore, the included studies exhibited high heterogeneity, which could affect the accurate assessment of the overall model performance and limit reliable inferences for specific subgroups. Variations in study design, patient characteristics, and data sources may have contributed to heterogeneity.

Although AI demonstrates promising outcomes in predicting severe COVID-19 mortality, it has ample scope for improvement. Continued research and development are necessary to enhance the sensitivity and specificity of prediction models. Moreover, AI applications of artificial intelligence extend beyond predicting COVID-19 prognoses. They are increasingly employed in diagnosing, screening, and managing other diseases, aligning with the objectives of tertiary prevention strategies. To promote the integration of AI and hospital digitalization, conducting high-quality, large-scale, multicenter studies is imperative. These studies advance the field of AI in healthcare and foster its effective implementation.

## 5 Conclusion

In summary, while artificial intelligence has shown promise in predicting severe mortality rates in COVID-19, the suitability of different models varies for specific populations, and there is still room for improvement in their predictive performance. In addition, The full applicability of these models in clinical practice remains uncertain. Therefore, ongoing research and development efforts are necessary to enhance the performance of these models. The application of AI extends beyond COVID-19; it is being utilized in diagnosing, screening, and managing other diseases, aligning with the objectives of tertiary prevention strategies. Therefore, conducting high-quality, large-scale, multicenter studies is imperative for advancing the field of AI in healthcare and ensuring its effective implementation.

## Data availability statement

The original contributions presented in the study are included in the article/supplementary material, further inquiries can be directed to the corresponding authors.

## Author contributions

CQ: Conceptualization, Data curation, Formal analysis, Investigation, Methodology, Resources, Software, Visualization, Writing – original draft. HM: Data curation, Formal analysis, Investigation, Resources, Software, Supervision, Writing – original draft. MH: Conceptualization, Methodology, Supervision, Validation, Visualization, Writing – original draft. XX: Conceptualization, Funding acquisition, Methodology, Project administration, Writing – review & editing. CJ: Conceptualization, Formal analysis, Funding acquisition, Methodology, Project administration, Visualization, Writing – original draft, Writing – review & editing.

## Footnotes

^1^ nih.gov

^2^ webofscience.com

^3^ Ovid
